# A Preliminary Test of Novelty-Facilitated Extinction in Individuals With Pathological Anxiety

**DOI:** 10.3389/fnbeh.2022.873489

**Published:** 2022-04-25

**Authors:** Shari A. Steinman, Joseph E. Dunsmoor, Zhamilya Gazman, Yael Stovezky, Olivia Pascucci, Justin Pomerenke, Elizabeth A. Phelps, Abby Fyer, H. Blair Simpson

**Affiliations:** ^1^Department of Psychology, West Virginia University, Morgantown, WV, United States; ^2^Department of Psychiatry and Behavioral Science, University of Texas at Austin, Austin, TX, United States; ^3^The Graduate Center, City University of New York, New York, NY, United States; ^4^New York State Psychiatric Institute, Division of Clinical Therapeutics, New York, NY, United States; ^5^Department of Psychiatry, Columbia University Medical Center, New York, NY, United States; ^6^The Steven A. Cohen Military Family Center, New York University Langone Health, New York, NY, United States; ^7^Department of Psychology, Harvard University, New York, NY, United States

**Keywords:** extinction, anxiety, obsessive-compulsive disorder, cognition, skin conductance

## Abstract

Studies with rodents and healthy humans suggest that replacing the expected threat with a novel outcome improves extinction and reduces the return of conditioned fear more effectively than threat omission alone. Because of the potential clinical implications of this finding for exposure-based anxiety treatments, this study tested whether the same was true in individuals with pathological anxiety (i.e., met DSM-5 diagnostic criteria for an anxiety disorder and/or obsessive-compulsive disorder (OCD). In this preliminary test of novelty-facilitated extinction, 51 unmedicated individuals with pathological anxiety were randomized to standard extinction (*n* = 27) or novelty-facilitated extinction (*n* = 24). Participants returned 24 h later to test extinction recall and fear reinstatement. Skin conductance responses (SCR) were the dependent measure of conditioned fear. Participants in both groups learned the fear association but variably extinguished it. Novelty did not facilitate extinction in this preliminary trial. Findings underscore the importance of translating paradigms from healthy humans to clinical samples, to ensure that new treatment ideas based on advances in basic neuroscience are relevant to patients.

## Introduction

Exposure therapy, in which individuals confront feared stimuli in a gradual manner to reduce fear, is a proven treatment for individuals with anxiety disorders and obsessive-compulsive disorder (OCD; Deacon and Abramowitz, 2004). However, following exposure therapy, while some individuals maintain their gains, many others (e.g., up to 62%) experience a return of fear (Craske and Mystkowski, 2006; Craske et al., 2008). There is a pressing need to determine ways to reduce relapse. Pavlovian fear conditioning and extinction is a valuable model to develop and test innovative treatments for psychopathology. Under standard extinction protocols, some studies (albeit not all) find that anxiety and OCD samples show deficits in learning and retaining extinction memories (Michael et al., 2007; Milad et al., 2013; Duits et al., 2016; Rabinak et al., 2017). The current study is a preliminary test of whether an augmented behavioral extinction strategy enhances fear extinction in individuals with pathological anxiety during a laboratory paradigm.

In typical lab-based extinction paradigms, participants learn that a conditioned stimulus (CS; e.g., a light) predicts an aversive unconditioned stimulus (US; e.g., a shock). The participants are then exposed to the CS multiple times without the US, leading to a reduction in defensive responses (often operationalized as “fear”) to the CS (e.g., reduced freezing in rodents, reduced skin conductance in humans). However, fear responses to the CS often return following a delay (Vervliet et al., 2013).

One method to enhance extinction is by replacing, rather than merely omitting, the expected aversive outcome. Dunsmoor et al. (2015) developed an extinction paradigm in which the US was replaced with a novel neutral stimulus (i.e., a tone). This procedure, referred to as novelty-facilitated extinction (NFE), was effective at decreasing return of fear responses 24-h after extinction in rats and healthy humans. This work has been replicated and extended in healthy humans: Lucas et al. (2018) demonstrated that NFE was effective at diminishing reinstatement (response to CS following return of aversive stimulus) in healthy humans, and Dunsmoor et al. (2019) found that NFE may lead to more durable extinction *via* activating the ventromedial prefrontal cortex during extinction trials. If NFE were to have similar effects in individuals with pathological anxiety it would provide specific suggestions on how to modify exposures to reduce relapse.

Associative learning literature provides potential explanations for why pairing a CS with a novel outcome might have advantages over standard extinction procedures. For example, instead of promoting a sense of safety, the omission of an expected threat in standard extinction may render the meaning of a CS ambiguous (Bouton, 2002). Pairing the CS with a novel stimulus might reduce ambiguity generated by threat omission alone. Further, because extinction is new associative learning (Pearce and Hall, 1980; Larrauri and Schmajuk, 2008), a novel but neutral outcome might generate a more durable association than a CS-no US association.

Reducing ambiguity may be particularly important for individuals with anxiety, given that these individuals respond to ambiguity differently than healthy individuals. Specifically, individuals with anxiety tend to interpret ambiguous information as threatening, rather than benign (Mathews and MacLeod, 2005). Additionally, these individuals are less able to tolerate uncertainty (Gentes and Ruscio, 2011). In healthy humans, intolerance of uncertainty is related to return of fear following a standard extinction paradigm, but not following an NFE paradigm (Dunsmoor et al., 2015; Lucas et al., 2018). Further, intolerance of uncertainty has been implicated in reduced extinction learning in healthy humans (Morriss et al., 2015, 2016). Together, observations suggest that reducing ambiguity *via* NFE may reduce post-extinction recall of fear.

The current study is a preliminary test of the effects of NFE in a sample of unmedicated individuals who met the criteria for an anxiety disorder or OCD. We predicted that participants in the NFE group would have less return and reinstatement of fear 24 h after extinction than participants in the standard extinction group.

## Methods and Materials

### Design

This study was conducted at the Anxiety Disorders Clinic, an outpatient research clinic, at the New York State Psychiatric Institute (NYSPI) and Columbia University Medical Center, and was approved by the NYSPI Institutional Review Board. Following informed consent, 67 unmedicated participants diagnosed with an anxiety disorder or OCD completed questionnaires and a differential fear conditioning paradigm followed by extinction. Participants were randomized to either standard extinction or NFE. Participants returned to the lab 24 h later to test post-extinction recall and reinstatement [skin conductance in response to conditioned stimulus (CS)].

### Participants

Participants were recruited *via* advertisements and referrals from physicians. Participants included adults aged 18–50 with a DSM-5 diagnosis of OCD, social anxiety disorder (SAD), generalized anxiety disorder (GAD), and/or specific phobia (SP). Diagnoses were made by trained clinicians (e.g., doctoral student with master’s degree, clinical psychologist, or psychiatrist) using a structured clinical interview (SCID; First and Spitzer, 1996). Participants were excluded if they used psychotropic medication in the last 4 weeks (8 weeks for selective serotonin reuptake inhibitors), had current diagnosis of major depressive disorder, or lifetime diagnosis of any psychotic disorder, bipolar disorder, or alcohol, or substance use disorder. Other exclusions included: acute suicidal risk; major medical or neurological problems that might interfere with study procedures or data interpretation or increased risk of participation (assessed *via* brief meeting with MD or Nurse Practitioner, e.g., cardiovascular disease, seizure disorder, head trauma); inability to refrain from caffeine (for 4 h) or nicotine (for 24 h) without withdrawal symptoms. Medication and drug use was established *via* self-report in the brief meeting with MD or Nurse Practitioner.

### Assessments

Following informed consent, participants completed a series of questionnaires, including the 21-item Beck Depression Inventory II (BDI-II; Beck et al., 1996) to assess depressive symptomatology, the 40-item State-Trait Anxiety Inventory (STAI; Spielberger et al., 1983) to assess both state (current, in the moment) and trait (how one typically feels) anxiety, and the Intolerance of Uncertainty Scale (IUS; scores range from 27 to 135; Buhr and Dugas, 2002) to assess response to uncertainty.

### Fear Acquisition, Extinction, and Recall

Participants completed the acquisition, extinction, and extinction recall, and reinstatement task over the course of 2 days. PsyLab (Contact Precision Instruments, Cambridge, MA, USA) was used to collect skin conductance data and run the acquisition, extinction, and recall tasks. On Day 1, two Ag/AgCl electrodes were affixed to the hypothenar eminence of the participants’ left hand, to assess skin conductance, and two Ag/AgCl electrodes were affixed to the participants’ right wrist, to deliver shocks. SignaGel Electrode Gel (Parker Laboratories, Inc., Fairfield, NJ, USA), a highly conductive saline gel, was used^1^. The shock was generated by the SHK1 Pain Stimulation Shocker (Contact Precision Instruments, Cambridge, MA, USA) and lasted 200 ms. A shock work-up was conducted to determine the level of shock that each participant found to be highly annoying, but not painful (Boucsein et al., 2012).

Following the work-up, participants were instructed to sit comfortably and pay attention to the images displayed on the screen. They were told they may or may not receive shocks, and that there would be an association between pictures and shocks, but they would need to learn it themselves. Participants wore headphones (Sennheiser PRO, Sennheiser electronic GmbH & Co. KG, Wennebostel, Wedemark, Germany) to block out noise and to deliver the novel tone to participants randomized to the NFE group.

Conditioned stimuli (CS) included two angry male faces (following Dunsmoor et al., 2015)^2^. Each trial included a CS displayed for 6 s, followed by a 12 s intertrial interval. The trial order was pseudorandomized so that no more than three trials of the same type occurred in a row. The conditioning session began with 10 habituation trials (five each of CS+ and CS-) to diminish initial orienting responses. This was followed by the first run of fear conditioning that included four CS+ trials that co-terminated with a shock to the wrist, seven CS+ trials unpaired with shock, and seven CS- trials. The second run of fear-conditioning included four CS+ trials paired with shock, eight CS+ trials unpaired with shock, and eight CS- trials. Conditioning was identical between groups. One rationale for using partial CS-US pairing is that continuous (100%) CS-US pairing rates can lead to a rapid decrease in conditioned responses (Grady et al., 2016) that would potentially obscure the effect of the extinction manipulation.

Following conditioning, the standard extinction group (EXT) underwent two runs of extinction that each included 10 CS+ trials unpaired with shock and 10 CS- trials. Following Dunsmoor et al. (2015), there was a very short pause (less than 1 min) between the first and second run of fear conditioning and extinction. For participants randomized to the NFE group, all CS+ extinction trials co-terminated with a low-volume 440-Hz tone for 1.5 s, delivered binaurally through headphones. Both the 200 ms shock (during acquisition for both groups) and the 1.5 s tone (during extinction for the NFE group) co-terminated with CS+. Thus, the onset of the tone during extinction preceded the onset of the shock during acquisition. The dB level of the tone was not recorded. The tone was meant to be perceptible but not loud or aversive. All of the subjects in the NFE condition were asked at the end of the experiment if they heard the tone, and all reported that they did.

**Figure 1:**
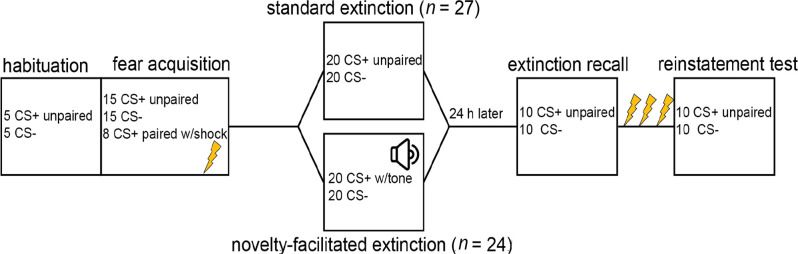
Graphic illustrating phases of the experiment. *Note*. Early and late fear acquisition were defined as the first and second run of conditioning, respectively. Early extinction was defined as the first run of extinction. Late extinction was defined as the last three CS+ and three CS- trials. Early recall and reinstatement were defined as the first three CS+ and three CS- trials.

Participants returned to the lab the following day. No new instructions were given for Day 2 that would indicate any departure from the procedures from the previous day. Electrodes were reattached and shock intensity was set at the level determined on Day 1. The recall included 10 CS+ trials unpaired with shocks (or tones) and 10 CS- trials. After these 20 CS trials, participants received three unsignaled shocks to the wrist to reinstate conditioned responses. The reinstatement test included 10 CS+ trials unpaired with shock and 10 CS- trials. See Figure 1 for an illustration of the phases of the experiment.

### Data Processing

Skin conductance responses (SCR) were collected using PsyLab (Contact Precision Instruments, Cambridge, MA, USA) at 500 Hz. Responses were calculated according to previous criteria (Dunsmoor et al., 2015) using a validated automated MATLAB (Mathworks, Inc., Natick, MA, USA) script (Green et al., 2014). CS+ trials paired with the shock during fear conditioning were not included in the analysis to avoid potential confounds introduced by the electrical shock. An SCR was considered related to the CS if the trough-to-peak deflection occurred within a 0.5–6.0 s time window starting at CS onset, if the responses lasted between 0.5 and 5.0 s, and if the response was greater than 0.2 microsiemens. If a response on a trial did not meet these criteria, it was scored as zero. Raw SCR values were square-root transformed to normalize the distribution. To account for different patterns of SCR over the course of learning, we divided the data into early and late phases. Early and late fear acquisition were defined as the first and second run of conditioning, respectively. Early extinction was defined as the first run of extinction. Late extinction was defined as the last 3 CS+ and 3 CS- trials, in order to capture responses toward the end of training (Dunsmoor et al., 2015). Early recall and reinstatement were likewise defined as the first three CS+ and three CS- trials, as prior research shows that extinction manipulations tend to affect early presentations of CS+ at extinction-retention tests (Milad et al., 2009; Schiller et al., 2010; Dunsmoor et al., 2015).

### Statistical Analysis

Following prior fear conditioning studies (Duits et al., 2016), repeated measures Analyses of Variance (ANOVAs) were run separately for each phase of the experiment (early acquisition, late acquisition, late extinction, early recall, early reinstatement). In each ANOVA, Group (EXT vs. NFE) was included as a between-subjects factor and stimulus (CS+, CS-) was included as a within-subjects factor. We followed-up significant two-way interactions with *t*-tests. Statistical significance was defined as *p* < 0.05.

## Results

### Demographics and Clinical Characteristics

Sixty-seven participants provided consent. Three were excluded from the analysis [did not return for the second day of study (*n* = 1), no longer met inclusion criteria (*n* = 1), technical errors (*n* = 1)]. An additional 13 participants were not included in the analysis due to a failure to show evidence of conditioned learning, as defined by no positive difference between mean SCRs to the CS+ vs. CS- during late conditioning (i.e., a difference of 0 or a greater mean SCRs to the CS- than CS+).

This resulted in 51 participants (60.80% female, *M* age = 25.23, *SD* age = 4.82, age-range = 18–41) included in the analysis: 27 in the EXT group and 24 in the NFE group. Participants in the two groups did not differ significantly in demographic or clinical characteristics (see Table 1 for *M*, *SD*, and tests for differences between groups).

**Table 1:** Demographics and clinical characteristics.

	Standard extinction (EXT) Mean (SD)*n* = 27 Mean (SD)	Novelty-facilitated extinction (NFE) *n* = 24 Mean (SD)	Statistical test for differences between groups
Age	24.33 (4.77)	26.24 (4.77)	*t*_(49)_ = 1.43, *p* = 0.159
Sex (*n*, %female)	15 (55.6%)	16 (66.7%)	*X*^2^_(1)_ = 0.66, *p* = 0.417
Na#x000EF;ve to Psychotropic Medication (*n*, %)	19 (70.4%)	18 (75.0%)	*X*^2^_(1)_ = 0.14, *p* = 0.712
Race (*n*, %)			*X*^2^_(4)_ = 1.40, *p* = 0.844
White	17 (63%)	16 (66.7%)	
Black	3 (11.1%)	2 (8.3%)	
Asian	3 (11.1%)	2 (8.3%)	
American Indian/	1 (3.7%)	0 (0%)	
Other	3 (11.1%)	4 (16.7%)	
Ethnicity (*n*, %)			*X*^2^_(1)_ = 0.04, *p* = 0.835
Hispanic or Latino	5 (18.5%)	5 (20.8%)	
Diagnosis (*n*, %)			*X*^2^_(4)_ = 1.94, *p* = 0.747
GAD	1 (3.7%)	2 (8.3%)	
OCD	4 (14.8%)	5 (20.8%)	
PD	1 (3.7%)	0 (0%)	
SAD	7 (25.9%)	7 (29.2%)	
More than one anxiety or OCD diagnosis	14 (51.9%)	10 (41.7%)	
Years of Education	14.81 (2.71)	15.92 (2.69)	*t*_(48)_ = 1.45, *p* = 0.153
STAI Trait	51.37 (9.32)	53.13 (9.88)	*t*_(49)_ = 0.65, *p* = 0.517
STAI State	41.81 (7.49)	46.75 (10.71)	*t*_(49)_ = 1.92, *p* = 0.060
BDI-II	14.11 (8.03)	14.38 (9.79)	*t*_(49)_ = 0.11, *p* = 0.916
IUS	75.22 (19.82)	81.08 (14.91)	*t*_(49)_ = 1.18, *p* = 0.243

*Note. GAD, Generalized Anxiety Disorder; OCD, Obsessive Compulsive Disorder; PD, Panic Disorder; SAD, Social Anxiety Disorder; STAI, State Trait Anxiety Inventory; BDI-II, Beck Depression Inventory II; IUS, Intolerance of Uncertainty Scale*.

As expected, STAI scores suggest elevated state (*M* = 44.14, *SD* = 9.39) and trait anxiety (*M* = 52.20, *SD* = 9.53) in both groups. These scores are approximately one and two standard deviations, respectively, above scores of healthy individuals (Spielberger et al., 1983). IUS scores reveal elevated levels of intolerance of uncertainty (*M* = 77.98, *SD* = 17.75); this mean score is approximately three standard deviations above a college sample without anxiety disorders (Freeston et al., 1994). BDI-II scores suggest minimal depression (*M* = 14.24, *SD* = 8.81).

The sample was free from psychotropic medications at the time of the study. Most participants (37 participants) had never used psychotropic medications. Of the 14 participants that had, weeks since the last dose ranged from 5 to 150.86 (*M* = 84.43, *SD* = 52.71), except for one participant who had a 2 mg dose of clonazepam 3 weeks prior to consent.

### Fear Acquisition, Extinction, Recall, and Reinstatement

**Figure 2:**
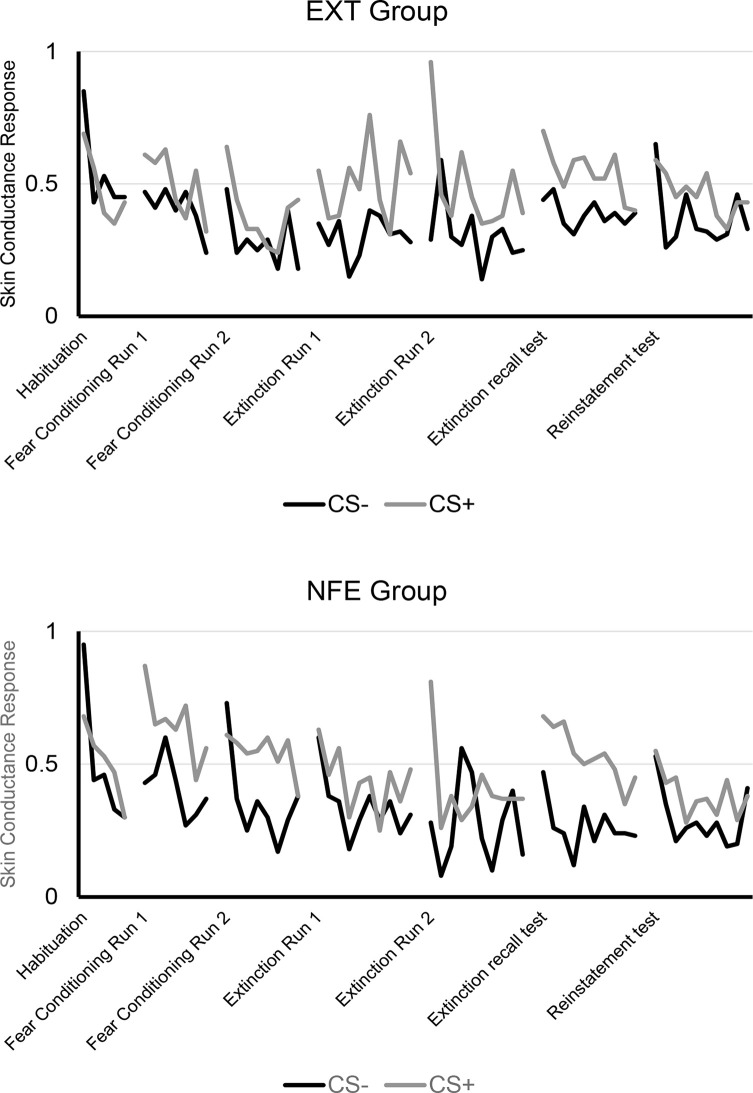
Trial by trial skin conductance response to CS+ and CS-, separated by group. *Note*. EXT, Standard Extinction; NFE, Novelty-Facilitated Extinction; CS, Conditioned Stimulus.

**Figure 3:**
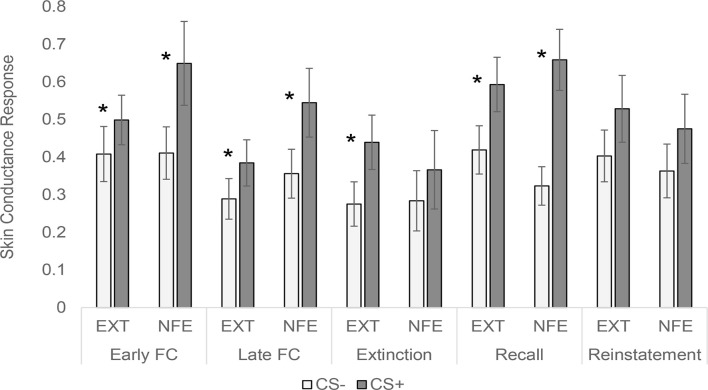
Skin conductance response to conditioned stimuli across experimental phases, separated by group. *Note*. EXT, Standard Extinction; NFE, Novelty-Facilitated Extinction; CS, Conditioned Stimulus. Error bars represent the standard error of mean. *p < 0.05 for paired sample *t*-tests comparing CS- to CS+ in each group.

See Figure 2 for trial by trial SCR to CS+ and CS-, separated by Group. See Figure 3 for means and standard errors of the means for each phase of the experiment, separated by Group. ANOVAs for early and late fear acquisition revealed significant effects of Stimulus (CS+, CS-), demonstrating that participants acquired conditioned SCRs to the CS+ compared to the CS- (early fear conditioning: *F*_(1,49)_ = 23.31, *p* < 0.001, $\eta_{p}^{2}$ = 0.32; late fear conditioning: *F*_(1,49)_ = 19.56, *p* < 0.001, $\eta_{p}^{2}$ = 0.29). There was no effect of Group for early fear acquisition (*F*_(1,49)_ = 0.49, *p* = 0.487, $\eta_{p}^{2}$ = 0.01), but results revealed a Stimulus by Group (NFE, EXT) interaction for early fear acquisition (*F*_(1,49)_ = 4.72, *p* = 0.035, $\eta_{p}^{2}$ = 0.09). Follow-up paired sample *t*-tests and visual inspection of means demonstrated that both groups successfully acquired conditioned fear during early fear conditioning (EXT: *t*_(26)_ = 2.40, *p* = 0.024, *d* = 0.25; NFE: *t*_(23)_ = 4.07, *p* < 0.001, *d* = 0.52), although the difference in response to CS+ to CS- was larger for the NFE group than the EXT group. For late fear acquisition, there was no effect of Group (*F*_(1,49)_ = 1.55, *p* = 0.219, $\eta_{p}^{2}$ = 0.03) or Stimulus by Group interaction (*F*_(1,49)_ = 2.10, *p* = 0.154, $\eta_{p}^{2}$ = 0.04), suggesting that during the second run of conditioning, participants successfully acquired similar levels of conditioned fear, regardless of group.

Contrary to expectations, the ANOVA for late fear extinction revealed a significant effect of Stimulus (*F*_(1,49)_ = 4.17, *p* = 0.047, $\eta_{p}^{2}$ = 0.08), indicating that SCRs remained elevated to the CS+ compared to the CS-. That is, participants still evinced heightened SCRs to the CS+ vs. the CS- in the last few trials of extinction, despite having been presented with 17 preceding CS+ trials during extinction without shock. Notably, there was no main effect of Group (*F*_(1,49)_ = 0.12, *p* = 0.724, $\eta_{p}^{2}$ = 0.002) or Stimulus by Group interaction (*F*_(1,49)_ = 0.46, *p* = 0.502, $\eta_{p}^{2}$ = 0.01). Overall, the extinction results suggest that conditioned fear was not extinguished in this anxiety sample, regardless of group.

Interpretation of the 24-h recall and reinstatement tests is complicated by the fact that neither group fully extinguished on Day 1. The ANOVA for 24-h recall revealed a significant effect of Stimulus (*F*_(1,49)_ = 38.63, *p* < 0.001, $\eta_{p}^{2}$ = 0.44), with no effect of Group (*F*_(1,49)_ = 0.03, *p* = 0.863, $\eta_{p}^{2}$ = 0.001) or stimulus by Group interaction (*F*_(1,49)_ = 3.88, *p* = 0.055, $\eta_{p}^{2}$ = 0.07). For the reinstatement test, there was no effect of stimulus (*F*_(1,49)_ = 4.02, *p* = 0.050, $\eta_{p}^{2}$ = 0.08), Group (*F*_(1,49)_ = 0.23, *p* = 0.637, $\eta_{p}^{2}$ = 0.01), or stimulus by Group interaction (*F*_(1,49)_ = 0.01, *p* = 0.910, $\eta_{p}^{2}$ < 0.001).

We also conducted an additional test to determine if there was an increase in differential responding from the end of extinction to the beginning of the 24-h recall test, despite incomplete extinction across participants. Specifically, we conducted a repeated measures ANOVA with Group (EXT vs. NFE) as a between subjects factor, and stimulus (CS+, CS-) and Phase (Late Extinction, Early Recall) as within subjects factors. Results revealed no effect of Group (*F*_(1,49)_ = 0.10, *p* = 0.752, $\eta_{p}^{2}$ = 0.002), Stimulus by Group interaction (*F*_(1,49)_ = 0.31, *p* = 0.580, $\eta_{p}^{2}$ = 0.01), Phase by Group interaction (*F*_(1,49)_ = 0.03, *p* = 0.870, $\eta_{p}^{2}$ = 0.001), or Phase by Stimulus by Group interaction (*F*_(1,49)_ = 2.67, *p* = 0.109, $\eta_{p}^{2}$ = 0.05). Additionally, there was no correlation between IUS and spontaneous recovery (as measured by SCR to CS+ during early trials of 24 h recall) in the entire sample (*r*_(49)_ = 0.10, *p* = 0.508), or in either group (EXT group: *r*_(25)_ = 0.23, *p* = 0.242; NFE group: *r*_(22)_ = −0.13, *p* = 0.545).

### Contingency Awareness

At the end of the experiment, we asked participants how often the shock followed the CS+ and CS- face. 86.3% of subjects correctly responded that the CS+ face was sometimes paired with shock, and 72.5% correctly responded that the CS- face was never paired with shock. We did not exclude subjects for incorrect contingency awareness, given that the question was asked retrospectively at the end of the experiment on Day 2.

## Discussion

To our knowledge, this preliminary study is the first to test the effects of novelty-facilitated extinction(NFE) compared to standard extinction (EXT) in 51 unmedicated participants diagnosed with an anxiety disorder or OCD. Neither group demonstrated within-session fear extinction. Further, novelty did not facilitate extinction. Although difficult to interpret (given the lack of extinction), novelty did not lead to reduced recall or reinstatement 24-h after extinction trials.

That participants in our study did not extinguish on Day 1 was unexpected given prior studies using the same paradigm in healthy individuals (Dunsmoor et al., 2015; Lucas et al., 2018). One explanation is that the CS used in our study (angry faces) may have been particularly anxiety-provoking for our sample, given that 70.6% of our sample met the criteria for SAD and individuals with social anxiety tend to interpret facial expressions in a more threatening way than non-anxious individuals (Mohlman et al., 2007; Yoon and Zinbarg, 2008). Another explanation could be a general abnormality in fear extinction in those with pathological anxiety. Indeed, several studies (albeit not all) suggest that individuals who meet the criteria for an anxiety disorder or OCD have extinction deficits compared to healthy control samples (Michael et al., 2007; Milad et al., 2013; Duits et al., 2016; Rabinak et al., 2017). Moreover, a meta-analysis by Duits et al. (2015) found a trend for increased response to CS+ vs. CS- during extinction in anxious individuals (defined as individuals that met criteria for DSM-IV anxiety disorders) compared to healthy controls. Another possibility is that tonic arousal is elevated, maintained, and carries over from acquisition into extinction in a clinically anxious sample. The result of impaired extinction learning could therefore be akin to the immediate extinction deficit described by Maren (2014), whereby elevated anxiety interferes with extinction learning processes. To address these different possibilities, future research testing novelty-facilitated extinction in a clinical sample should include a healthy control group, increase the number of extinction trials, use stimuli other than angry faces, and test the effect of immediate vs. delayed extinction.

The inability of the NFE paradigm to produce similar effects in clinically anxiety participants as shown previously in healthy adults (Dunsmoor et al., 2015, 2019; Lucas et al., 2018) raises important questions on how to leverage insights into the mechanisms of fear extinction to improve exposure-based therapy. There is an excitement that laboratory-based approaches centered on inhibitory learning and memory updating can translate to the clinic (Fullana et al., 2020). This includes techniques such as counter conditioning (Keller et al., 2020), memory reconsolidation updating, and pharmaceutical adjuncts to enhance learning during psychotherapy (Phelps and Hofmann, 2019). However, many issues remain in translating basic research in healthy adults to clinical populations, and much more work is needed to discover how to optimize behavioral protocols to yield similar effects in people with diagnosed anxiety disorders and OCD. Future designs should consider more extensive extinction-based training (e.g., multiple sessions) and be attentive to individual participants’ ability to successfully extinguish conditioned fear as a precondition to test the return of fear.

This study has several strengths: an unmedicated, transdiagnostic sample with pathological anxiety, and the use of an established laboratory paradigm (Dunsmoor et al., 2015). Results should be considered in light of the limitations described above as well as the fact that our sample was predominantly white and non-Hispanic. Despite limitations, our preliminary findings provide insight into methodological considerations for future tests of novelty-facilitated extinction, and data can be included in future meta-analyses.

In summary, our preliminary data is in line with prior findings that demonstrate extinction deficits in those with anxiety disorders and OCD, and extend these findings to suggest that such individuals may process novel information differently than healthy individuals (though replication with a diagnosed vs. healthy sample is needed). In addition, our data highlight the importance of testing whether basic and clinical neuroscience findings gleaned in healthy populations translate to clinical samples. This will ensure that new treatment ideas based on basic neuroscience advances are indeed relevant for patients.

## Data Availability Statement

The raw data supporting the conclusions of this article will be made available by the authors, without undue reservation.

## Ethics Statement

The studies involving human participants were reviewed and approved by NYSPI Institutional Review Board. The patients/participants provided their written informed consent to participate in this study.

## Author Contributions

SS: funding acquisition, conceptualization, formal analysis, investigation, writing—original draft, writing—review and editing. JD: conceptualization, methodology, resources, data curation, writing—review and editing. ZG: software, investigation, and resources. YS: investigation and project administration. OP and JP: project administration. EP: resources and supervision. AF: resources, supervision, writing—review and editing. HBS: resources, supervision, writing—review and editing. All authors contributed to the article and approved the submitted version.

## Conflict of Interest

In the past three years, HBS reports royalties from Cambridge University Press and UpToDate, Inc. and research support for an industry-sponsored trial of an investigational drug for obsessive-compulsive disorder from Biohaven. She also receives a stipend for her role as Associate Editor of JAMA Psychiatry. The remaining authors declare that the research was conducted in the absence of any commercial or financial relationships that could be construed as a potential conflict of interest.

## Publisher’s Note

All claims expressed in this article are solely those of the authors and do not necessarily represent those of their affiliated organizations, or those of the publisher, the editors and the reviewers. Any product that may be evaluated in this article, or claim that may be made by its manufacturer, is not guaranteed or endorsed by the publisher.

## Footnotes

^1^Guidelines for assessing electrodermal activity (Boucsein et al., 2012) recommend against using non-isotonic gels, such as SignaGel, as it may artificially inflate skin conductance. However, the differential nature (CS+ vs. CS-) of our outcome variables likely protects the validity of our results.

^2^The task design was similar to the design from Dunsmoor et al. (2015). However, we added additional trials to the extinction session. This was done a priori with the assumption that anxiety patients would need more trials to fully extinguish. Indeed, despite this effort, many anxiety patients still failed to show evidence of extinction of SCRs by the end of the session. Angry faces were selected (vs. neutral sensory stimuli, such as colored squares) as CS because they are “fear relevant,” and can be used to more reliably assess the effects of an enhanced extinction technique, as using a purely neutral CS can lead to rapid extinction. A tone was selected as the novel outcome because subjects would clearly process it as a concrete outcome that occurs at the moment in time that the shock is expected.
